# Poly(arylene ether nitrile) Based Dielectrics with High Energy Storage Properties: A Review

**DOI:** 10.3390/nano15090696

**Published:** 2025-05-05

**Authors:** Yongxian Liu, Guangjun Liu, Yayao Jiao, Zaixing Wang, Shumin Bao, Xiufu Hua, Lingling Wang, Bo Tang, Zhiyuan Xiong, Renbo Wei

**Affiliations:** 1 School of Chemical Engineering, Northwest University, Xi’an 710069, China; liuyongxian@stumail.nwu.edu.cn (Y.L.); 2023115054@stumail.nwu.edu.cn (G.L.); jiaoyy@stumail.nwu.edu.cn (Y.J.); wangzaixing@stumail.nwu.edu.cn (Z.W.); baoshumin@stumail.nwu.edu.cn (S.B.); wangll@nwu.edu.cn (L.W.); 2 Yangtze Delta Region Institute, Tsinghua University, Jiaxing 314006, China; 3 School of Materials Science & Engineering, Chongqing University of Technology, Chongqing 401320, China; 4 School of Light Industry and Engineering, South China University of Technology, Guangzhou 510006, China

**Keywords:** PEN composites, dielectric constant, breakdown strength, energy density

## Abstract

Polymer-based nanocomposites have demonstrated significant strategic value in dielectric energy storage systems due to their tunable high energy density and rapid charge–discharge efficiency. Poly(arylene ether nitrile) (PEN), owing to its superior thermal stability, high mechanical strength, chemical corrosion resistance, and outstanding dielectric properties, exhibits distinct advantages in the field of high-performance dielectric energy storage devices. This review focuses on key strategies for enhancing the dielectric energy storage performance of PEN-based composites, emphasizing molecular engineering approaches, microstructural design, the multiscale interface regulation mechanisms within composite systems, and the optimization of the dielectric constant (*ε*_r_) and breakdown strength (*E*_b_) through thermal stretching. Furthermore, the potential of PEN-based polymer composites in energy storage devices is highlighted, and future research directions are proposed, including the establishment of a dynamic balance mechanism between dielectric/insulating properties and the development of novel composite systems that offer both high energy storage density and stability. These advancements will provide the material foundation for the miniaturization and intellectualization of advanced pulse power equipment.

## 1. Introduction

The global transition towards cleaner and more efficient energy architectures has positioned the development of advanced energy storage technologies with both high power density and operational reliability as a pivotal driver for the energy revolution [1,2,3,4,5]. As one of the core components for next-generation energy storage devices, dielectric capacitors have demonstrated irreplaceable value in pulsed power applications including smart grid frequency regulation [6,7], electromagnetic weapon systems [8,9], and new energy electric vehicles owing to their ultra-fast charge–discharge characteristics (on the μs timescale) and exceptional power density (~10^8^ W/kg) [10,11,12,13]. Among various dielectric materials for these capacitors, polymer-based composite systems have attracted significant attention due to their superior processability, high *E*_b_, and excellent mechanical flexibility [14,15,16,17,18,19]. Notably, poly(arylene ether nitrile) (PEN), an emerging high-performance engineering plastic, has emerged as a research hotspot due to its unique aromatic ether-nitrile synergistic effects [20,21,22,23,24]. This molecular structure endows PEN with remarkable *ε*_r_ and superior solution/melt-processing capabilities [25,26,27,28], demonstrating potential to surpass the performance limitations of conventional poly(vinylidene fluoride) (PVDF)-based materials, particularly in high-temperature dielectric applications [29,30].

As a semi-crystalline polymer featuring aromatic ether linkages and strongly polar nitrile groups (–C≡N) in its structure [31], PEN exhibits exceptional comprehensive properties through molecular engineering: The rigid skeleton constructed by aromatic ether units ensures ultrahigh glass transition temperature (T_g_ > 150 °C), thermal decomposition temperature (T_d_ > 450 °C) and melting point (T_m_ > 330 °C), guaranteeing thermal stability under extreme operating conditions [32]. Meanwhile, the high dipole moment from nitrile groups significantly enhances chain polarization capability, resulting in a higher *ε*_r_ (*ε*_r_ ≈ 3.8 at 1 kHz) than other polymers such as polyimide [33]. This unique ‘rigid-flexible balance’ characteristic renders PEN particularly promising for energy storage applications under high-temperature and high-electric-field conditions [34]. Furthermore, compared with other high-performance polymers such as polyimide (PI), polyetherimide (PEI), and polyetherketone (PEK), PEN demonstrates enhanced solution processability and melt-processing capabilities attributable to its pendant cyano groups [35,36]. However, current studies reveal that the energy storage density of pure PEN (U_e_ ≈ 0.78 J/cm^3^) remains inadequate for next-generation power systems’ demands on dielectric capacitors, resulting in an urgent need for performance breakthroughs through innovative material design and processing technologies [37].

Energy storage density serves as a key parameter for evaluating the energy storage performance of dielectric materials. When subjected to an external electric field, dielectric materials undergo polarization phenomena that generate induced charges on the electrode plates, enabling energy storage. The energy storage density of dielectric materials can be calculated using Equation (1) [38]:

*U_e_* = ∫*E*d*D* (1)

where *U_e_* denotes the storage density, *E* represents the applied field strength, and *D* corresponds to the electric displacement. *D*, characterizing the material’s response to electric fields, equals the surface charge density of parallel-plate capacitors. As governed by Maxwell’s equations describing fundamental electromagnetic principles, the relationship between electric displacement and applied field can be expressed as Equation (2) [39]:

*D* = *ε*_0_*ε*_r_*E* (2)

For linear dielectrics such as PEN where *ε*_r_ remains field-independent, the energy density formula simplifies to Equation (3) [40]:

*U*_e_ = 0.5*ε*_0_*ε*_r_*E*_b_^2^ (3)

where *ε*_0_ is the vacuum permittivity (8.85 × 10^−12^ F/m). This formulation reveals that energy storage density is dependent on *ε*_r_ and quadratic *E*_b_. Consequently, enhancing these two parameters constitutes the principal pathway for energy storage performance improvement. Current dielectric optimization strategies focus on two principal approaches: intrinsic molecular polarization enhancement and composite synergy of extrinsic polarization. Molecular engineering approaches that directionally introduce high-polarity functional groups (e.g., cyano and fluoro groups) into the PEN backbone can substantially enhance dipolar orientation capabilities while maintaining high insulation properties [7,12]. The extrinsic composite strategy involves introducing organic fillers, perovskite ceramic nanoparticles (e.g., barium titanate), or conductive fillers (e.g., graphene) into the PEN matrix to generate synergistic dielectric enhancement through interfacial polarization and micro-capacitance effects. Notably, surface functionalization techniques have emerged as critical approaches to enhance filler dispersion and optimize interfacial dielectric compatibility [15,20], which is essential for balancing dielectric response and loss characteristics in composite systems.

Enhancement of *E*_b_ requires coordinated breakthroughs in intrinsic defect mitigation and electric field distribution regulation. Molecular design strategies, including co-polymerization modification for chain rigidity enhancement and cross-linked network construction, can effectively suppress charge carrier migration and space charge accumulation [3,22]. Concurrently, multilayered composite architecture design or incorporation of 2D insulating fillers (e.g., boron nitride, BN) can physically block electrical treeing propagation pathways and homogenize electric field distribution. The integration of these multi-scaled modification strategies provides new possibilities for achieving both high *E*_b_ and superior energy storage efficiency. The uniaxial hot-stretching technique leverages synergistic effects between molecular chain alignment and in-plane filler orientation, simultaneously enhancing crystallization (reducing defect density) and establishing anisotropic conductive pathways [6,27]. This innovative approach demonstrates unique advantages in the simultaneous optimization of *ε*_r_ and *E*_b_, offering new pathways to overcome performance limitations of conventional modification methods.

This review systematically summarizes recent advancements in PEN-based dielectric materials, focusing on elucidating optimization mechanisms for *ε*_r_ and *E*_b_. We firstly expound the intrinsic polarization enhancement through molecular engineering strategies, then discuss multi-scaled interface regulation mechanisms in filler composite systems, and conclusively analyze microstructure design principles for electric field optimization. Special emphasis is placed on emerging technologies like uniaxial hot-stretching, with detailed discussion on their dual-enhancement mechanism for *ε*_r_ and *E*_b_ via synergistic molecular chain orientation and filler alignment. Finally, we outline current challenges in PEN-based composites and propose multi-scaled structural design as a promising research direction, offering fundamental insights for developing high-performance PEN-based energy storage materials.

## 2. Strategic Framework for Enhancing Dielectric Constant of PEN

This section methodically elaborates three principal strategies to improve the dielectric constant of PEN for high energy storage performance: molecular structure design, composite system construction (organic/ceramic/conductive fillers in PEN matrix), and uniaxial orientation hot-stretching technology.

### 2.1. Polymer Molecular Structure Design

The molecular architecture of polymers critically governs their dielectric energy storage characteristics. Particularly, higher concentrations of polar functional groups (e.g., cyano (–CN), ether linkages (–O–), hydroxyl (–OH)) in polymer chains generally correlate with enhanced *ε*_r_. However, excessive polar groups may compromise *E*_b_ [31], owing to localized polarization-induced electric field concentration effects under applied fields. Therefore, engineering PEN molecular structures with optimized polar group density is essential for achieving superior dielectric energy storage performance. Furthermore, the chain rigidity and crystallization of PEN also significantly influence its dielectric properties. Enhanced chain rigidity contributes to improved *E*_b_, while controlled crystallization optimizes the *ε*_r_-*E*_b_ trade-off. How to achieve a rational distribution of polar groups, control the rigidity of molecular chains, and optimize the crystallization in PEN through molecular design remains a key challenge in current research [9]. This section will explore strategies to enhance the dielectric performance of PEN through molecular structure design.

**Figure 1:**
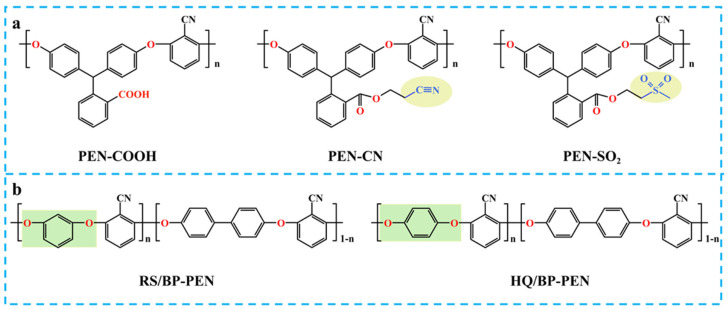
The molecular structural formulas of (**a**) DGP [22] and (**b**) BP-PEN [20].

Recent advances in molecular structure design for PEN side-chain engineering have emerged as a research frontier for *ε*_r_ enhancement. As demonstrated by Huang et al., strategic incorporation of methanesulfonyl (–SO_2_CH_3_) and cyanoethyl (–CH_2_CH_2_CN) groups combined with phenolphthalin (PPL) non-planar structures enabled the successful synthesis of novel polar glass polymers (DGPs, Figure 1a) [22]. These materials exhibit high *ε*_r_ (4.3 for PEN-CN and 4.5 for PAEN-SO_2_) with low loss tangents (tanδ < 0.01), while maintaining high glass transition temperatures (T_g_ > 200 °C). This phenomenon originates from the strong polarity of cyano (–CN) and sulfonyl (–SO_2_–) groups, which were strategically incorporated into polymer side-chains, significantly amplifying molecular polarity and polarization capability through enhanced dipole moment interactions.

Complementary work by Chen et al. focused on main-chain optimization via bisphenol monomer engineering [20]. By modulating hydroquinone (HQ) and resorcinol (RS) ratios in 4,4′-biphenol (BP)-based PEN systems (Figure 1b), they achieved *ε*_r_ enhancement up to 4.1 at 1 kHz (2.8-RS/BP-PEN). This improvement stems from structural disorder induced by HQ/RS incorporation, which reduces crystallization while increasing chain polarity. Notably, RS-modified PEN demonstrated reduced *T*_g_ with preserved chain mobility at elevated temperatures, which is beneficial for high energy storage performance.

**Figure 2:**
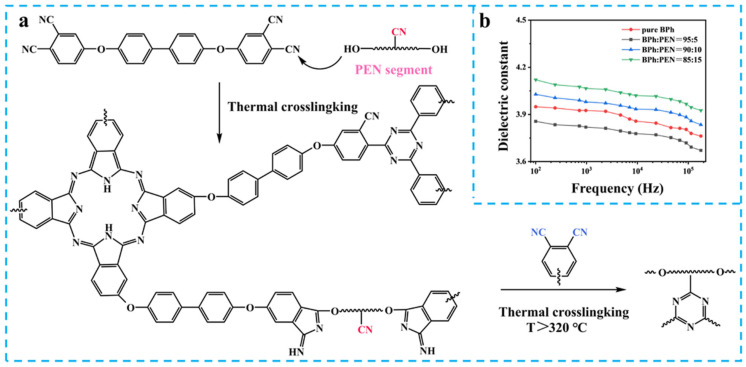
(**a**) The synthetic route and (**b**) *ε*_r_ of BPh/PEN [23].

Pioneering work by Huang et al. introduced 4,4′-bis(3,4-dicyanophenoxy) biphenyl (BPh)/PEN-OH copolymers featuring dual crosslinking mechanisms: nitrile polymerization and triazine ring formation (Figure 2a) [23]. The resulting thermosetting networks exhibited stable *ε*_r_ (>4.1) across broad frequencies (100 Hz–200 kHz) with excellent thermal adaptability, attributed to optimized crosslinking density and hierarchical polarization behavior (Figure 2b). This molecular engineering approach establishes new paradigms for developing high-performance dielectric polymers through controlled chemical architecture design.

These findings establish that backbone engineering enables effective enhancement of PEN dielectric constant through three key strategies: (1) incorporating strong polar groups (e.g., cyano (–CN), sulfonyl (–SO_2_–)) to amplify molecular polarization [8]; (2) modulating monomer architecture and stoichiometry to optimize the chain polarity–crystallization balance [17]; (3) constructing crosslinked networks to enhance material stability and dielectric response. Crucially, these approaches not only elevate *ε*_r_ but also achieve suppressed dielectric loss tangent (tan*δ*) through controlled polar group dynamics.

### 2.2. Organic Filler/PEN Composites

Conventional homopolymer dielectrics are intrinsically limited by low *ε*_r_ (*ε*_r_ < 4.0), failing to meet the escalating energy density demands of advanced electronics. This challenge has driven the emergence of organic composite strategies as a new frontier for PEN-based dielectric optimization. By integrating high *ε*_r_ organic fillers, these systems synergistically optimize interfacial polarization and dielectric response characteristics through tailored phase interactions. This section critically reviews breakthroughs in PEN composites with archetypal organic fillers: PVDF, conductive polymers (e.g., polyaniline, PANI), metallic phthalocyanine (MPc) polymer, etc., with emphasis on filler–matrix interaction mechanisms.

**Figure 3:**
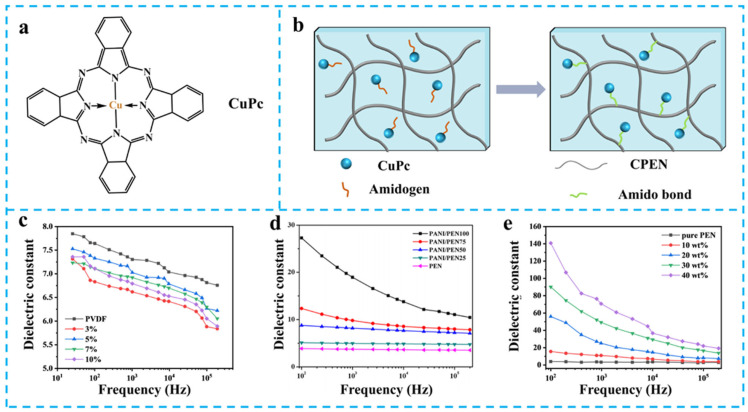
(**a**) The molecular structure of CuPc; (**b**) Schematic diagrams of the formation of chemical bond in the PEN/CuPc films [46]; The *ε*_r_ of (**c**) PVDF/PEN [41], (**d**) PANI/PEN, (**e**) CuPc/PEN [46]. Picture (**d**) was adapted with permission from Ref. [42]. Copyright 2016 Spring Nature.

Pioneering work by Long et al. demonstrated PVDF/PEN blends prepared via solution casting [41]. With PVDF loading reaching 95 wt%, the composite exhibited exceptional dielectric stability (*ε*_r_ gradually decreasing from 7.2 to 6.0 across 25 Hz–1 MHz) (Figure 3c). This weak frequency dependence originates from micro-capacitor networks formed by alternating PVDF-rich domains (high *ε*_r_ phase) and PEN domains (low *ε*_r_ phase), effectively suppressing interfacial charge accumulation. Notably, the parallel-series capacitor analogy explains the inhibited dielectric relaxation phenomena. However, excess PVDF (>95 wt%) disrupts phase-separation periodicity, leading to escalated tan*δ* due to disordered charge transport pathways.

The strategic incorporation of conductive polymer fillers demonstrates superior dielectric enhancement compared to physical blending approaches, primarily through amplified interfacial polarization effects. Related work by Wei et al. employed in situ doping to integrate sulfuric acid-modified polyaniline (PANI) into the PEN matrix [42]. At 10 wt% PANI loading, the composite achieved a remarkable *ε*_r_ of 18.5 at 1 kHz (500% enhancement vs. pure PEN) (Figure 3d), governed by Maxwell–Wagner–Sillars (MWS) polarization. Percolating PANI networks create interfacial charge polarization zones due to dielectric mismatch with the PEN matrix. Notably, the composite maintained *ε*_r_ stability (<5% fluctuation) across 25–180 °C and survived in 10 thermal cycles without the obvious increment of tan*δ* (tan*δ* ≈ 0.25), attributed to the organic nature of PANI, which suppresses filler agglomeration and charge migration.

Phthalocyanines (Pc), particularly copper phthalocyanine (CuPc, Figure 3a), have emerged as promising dielectric fillers owing to their tunable 18-π-electron conjugated systems [43,44,45]. Yang et al. fabricated PEN-COOH/CuPc nanocomposites via solution casting, where –COOH/–NH_2_ hydrogen bonding and amide linkages ensured homogeneous dispersion (Figure 3b) [46]. At 40 wt% CuPc loading, the *ε*_r_ reached 45 at 1 kHz (1000% improvement) (Figure 3e), while chemical bonding effectively suppressed leakage currents through interfacial defect passivation [47,48,49].

These studies reveal synergistic dielectric regulation mechanisms: (1) insulator–insulator systems (e.g., PVDF/PEN): dielectric enhancement arises from dimensional confinement effects in phase-separated architectures, where micro-capacitor networks redistribute bound charges. (2) Insulator–conductive systems (e.g., PANI/PEN): MWS polarization dominates, with percolation threshold-dependent dielectric response. (3) Interfacial engineering: covalent/non-covalent interactions (hydrogen bonds, π–π stacking) minimize interfacial defects, enabling concurrent high-*ε*_r_ and low-tan*δ* performance.

### 2.3. Ceramic Filler/PEN Composites

Ceramic fillers such as barium titanate (BaTiO_3_, *ε*_r_~2000) and copper calcium titanate (CuCa_3_Ti_3_O₁_2_, CCTO, *ε*_r_~10⁴) demonstrate superior polarization enhancement capabilities in PEN composites due to their high *ε*_r_ [50,51]. However, these inorganic fillers suffer from intrinsic agglomeration tendencies and poor interfacial compatibility with the PEN matrix, often leading to interfacial defects and localized electric field distortions. Recent breakthroughs in filler morphology engineering and interfacial modification have addressed these critical challenges.

**Figure 4:**
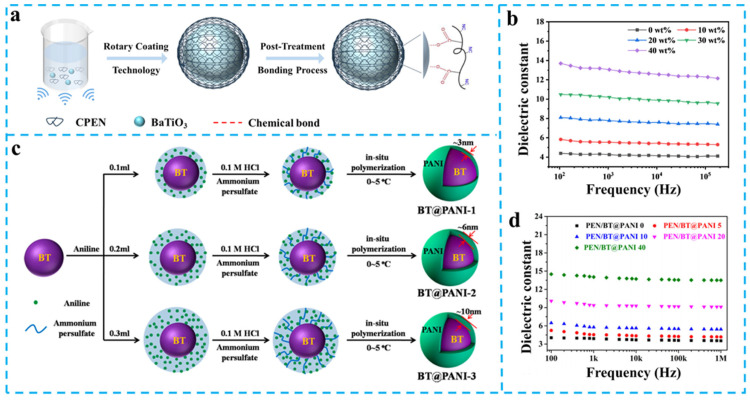
(**a**) Schematic diagram of the fabrication of core–shell structured CPEN@BaTiO_3_ nanoparticles [53]; (**b**) The *ε*_r_ of CPEN@BaTiO_3_/PEN [53]; (**c**) Schematic diagram of core–shell structured BT@PANI nanoparticles; (**d**) The *ε*_r_ of BT@PANI/PEN. Pictures (**c**,**d**) were adapted with permission from Ref. [54]. Open access under the terms of the Creative Commons Attribution License (CC BY 4.0).

Early-stage investigations focused on physical dispersion methods for homogeneous filler distribution. As exemplified by Tang et al., continuous ultrasonication dispersion technology was employed to fabricate BaTiO_3_/PEN nanocomposite films [52]. At 40 wt% filler loading, the composite achieved *ε*_r_ = 12.5 (220% enhancement vs. pure PEN). Although ultrasonication effectively suppressed BaTiO_3_ agglomeration without using dispersant, it failed to resolve fundamental interfacial incompatibility issues. To overcome this limitation, advanced surface grafting techniques were developed. A breakthrough involved constructing CPEN@BaTiO_3_ core–shell architectures via spin-coating and post-treatment processes [53]. Carboxylated PEN (CPEN) formed 4–7 nm coating layers on BaTiO_3_ through monodentate coordination, improving dispersion and interfacial compatibility. This innovation elevated *ε*_r_ to 13.1 at 40 wt% loading (Figure 4a,b), while maintaining tan*δ* < 0.02 at 1 MHz through defect passivation effects.

**Figure 5:**
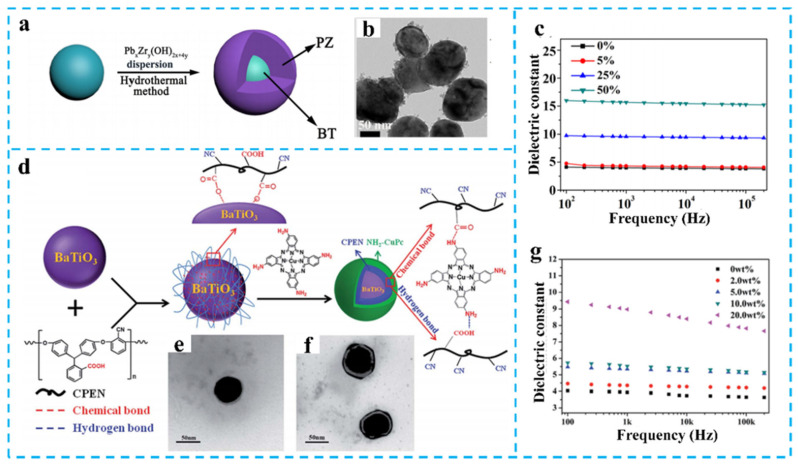
(**a**) Scheme for the fabrication of PZ@BT nanoparticles; (**b**) TEM image of PZ@BT; (**c**) The *ε*_r_ of PZ@BT/PEN; (**d**) The preparation of the CPEN-f-BT@CuPc nanoparticles; TEM images of CPEN-f-BT (**e**) and CPEN-f-BT@CuPc (**f**); (**g**) The *ε*_r_ of CPEN-f-BT@CuPc/PEN. Picture (**a**–**c**) were adapted with permission from Ref. [55]. Copyright 2018 Spring Nature. Picture (**d**–**g**) were adapted with permission from Ref. [56]. Open access under the terms of the Creative Commons Attribution License (CC BY 4.0).

Recent breakthroughs demonstrate enhanced interfacial polarization through conductive polymer coating strategies. Wei et al. developed BT@PANI core–shell architectures via in situ polymerization [54], achieving controlled PANI coating thickness (3–10 nm) on BaTiO_3_ (Figure 4c). At 40 wt% loading, the composite exhibited *ε*_r_ = 14.0 at 1 kHz (260% enhancement vs. pure PEN, Figure 4d), attributed to intensified interfacial polarization. A more advanced PZ@BT system with 20 nm PZ shells was synthesized through modified hydrothermal methods (Figure 5a,b) [55]. This ferroelectric/antiferroelectric hetero-structure demonstrated *ε*_r_ = 15.6 at 50 wt% loading (380% improvement, Figure 5c), resulting from ferroelectric-relaxor coupling that modulates band structures while suppressing charge migration, and enabled concurrent *ε*_r_ enhancement and loss reduction.

Building on the mono-layered structure, the team led by Liu et al. proposed a gradient dielectric design concept, successfully preparing core–shell–shell structured nanoparticles (CPEN-*f*-BT@CuPc, Figure 5d,f) by grafting amino copper phthalocyanine (NH_2_-CuPc) onto CPEN modified BaTiO_3_ [56]. The bi-layered interfacial system constructed using a combination of spin-coating and ultrasonic dispersion techniques enhanced the *ε*_r_ to 9.0 at a filler loading of 20 wt% (Figure 5g). The structure synergistically enhanced interfacial compatibility through the inner CPEN layer and formed a charge trap layer with the outer CuPc, effectively alleviating the dielectric mismatch between the filler and matrix, thereby enabling a linear increase in *ε*_r_ under low loss conditions.

**Figure 6:**
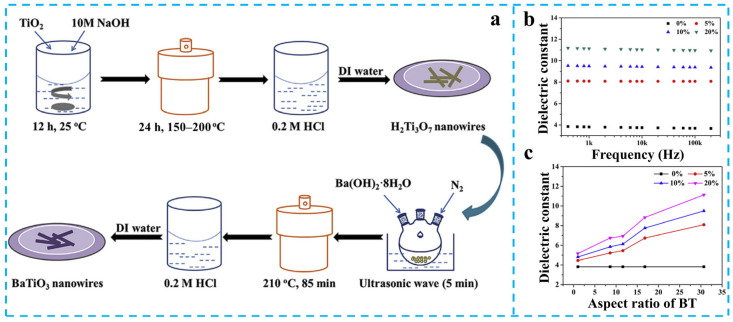
(**a**) The procedure for the preparation of BT-NWs; (**b**) The *ε*_r_ of BT-NWs/PEN; (**c**) The *ε*_r_ of BT-NWs/PEN composites with variation of aspect ratio of BT at 1 kHz. Picture (**a**–**c**) were adapted with permission from Ref. [57]. Copyright 2019 Elsevier.

**Figure 7:**
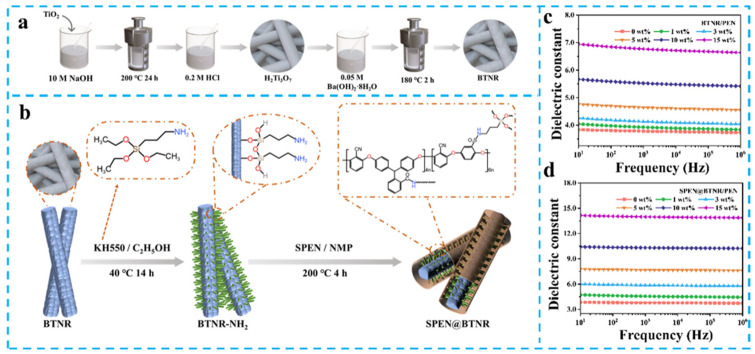
(**a**) Scheme for synthesizing BTNR; (**b**) Synthesis route of SPEN@BTNR; The *ε*_r_ of (**c**) BTNR/PEN and (**d**) SPEN@BTNR/PEN. Picture (**a**–**c**) were adapted with permission from Ref. [59]. Copyright 2024 Springer Nature.

Although conventional particulate BaTiO_3_ offers facile processability due to its isotropic nature, its limited specific surface area and inherent agglomeration tendencies restrict dielectric enhancement in PEN composites. To address these problems, Tu et al. developed barium titanate nanowires (BT-NWs) with controlled aspect ratios (8.6, 11.6, 16.8, and 30.7) via a two-step hydrothermal method (Figure 6a) [57]. SEM characterization confirmed homogeneous BT-NWs dispersion in the PEN matrix without agglomeration. Dielectric measurements revealed progressive *ε*_r_ enhancement with increasing BT-NWs loading and aspect ratio, achieving *ε*_r_ = 11.1 at 1 kHz (300% vs. pure PEN, Figure 6b,c) with 20 wt% loading and aspect ratio 30.7. This originates from high-aspect-ratio BT-NWs establishing continuous polarization pathways through intensified interfacial polarization. Complementary work by Zhang et al. engineered surface-aminated BT nanorods (BTNR) using KH550, subsequently coated with sulfonated PEN (SPEN) to form SPEN@BTNR fillers (Figure 7a,b) [58,59]. The chemical similarity between SPEN and PEN matrix ensured excellent dispersion, yielding *ε*_r_ = 14.0 at 15 wt% loading (1 kHz, Figure 7c,d). This represents a 367% enhancement over pure PEN, attributed to optimized interfacial charge distribution and reduced leakage currents.

These studies demonstrate that 1D nanostructured engineering combined with interface modification can enhance contact area, dispersion of filler in the PEN matrix, thus increasing effective polarization interfaces, and prevent filler agglomeration-induced defects [60,61]. The synergistic effects enable simultaneous enhancement of *ε*_r_ (*ε*_r_ > 300%) and energy storage density [62,63,64].

### 2.4. Conductive Filler/PEN Composites

The advancement of conductive filler-modified PEN composites centers on two principal material systems: metallic fillers (Ag, Ni, Al) and carbon nanomaterials (carbon nanotubes, graphene derivatives, etc.) [65,66,67]. Metallic systems leverage intrinsic high conductivity to construct high *ε*_r_ architectures, while carbon-based counterparts exploit unique electron polarization and percolation effects. Notably, conductive fillers achieve 3–5 orders of magnitude *ε*_r_ enhancement at low loading (<5 wt%) through micro-capacitor formation and interfacial polarization, outperforming traditional ceramic fillers. However, persistent challenges include leakage currents near percolation thresholds and van der Waals force-induced charge accumulation at filler interfaces, necessitating advanced dispersion control strategies.

Pioneering work by Li et al. established Ag nanoparticle/PEN composite films via in situ reduction as a model system [68]. Systematic investigation revealed concentration-dependent morphological transitions: <1.0 wt% AgNO_3_ yields 80–110 nm Ag nanospheres, while >2.0 wt% AgNO_3_ results in nanorods longer than 300 nm. Remarkably, these unmodified Ag fillers achieved homogeneous dispersion through optimized reduction kinetics, enabling *ε*_r_ = 5.8 at 2.0 wt% loading (45% enhancement vs. pure PEN). This breakthrough provides critical insights into interface engineering for metal/polymer dielectric systems.

**Figure 8:**
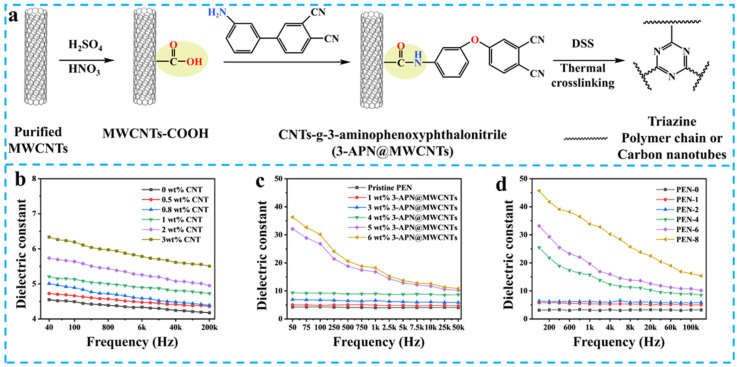
(**a**) Synthesis route for 3-APN@MWCNTs and the crosslinking of cyano groups [71]; The *ε*_r_ of CNTs/PEN (**b**) [69], 3-APN@MWCNTs/PEN (**c**) [70], and 3-APN@MWCNTs/PEN after crosslinking (**d**) [71].

Carbon nanotubes (CNTs), as representative one-dimensional nanofillers, exhibit exceptional dielectric polarization responses due to their sp^2^-hybridized delocalized π-electron system. As demonstrated by Zheng et al. [69], incorporation of 3 wt% multi-walled carbon nanotubes (MWCNTs) enhanced the *ε*_r_ of biphenyl-typed PEN from 4.3 to 6.1 at 1 kHz (Figure 8b). However, the concomitant increase in loss factor from 0.010 to 0.026 revealed compatibility issues arising from filler aggregation in conventional blending methods. Jin et al. developed amino-functionalized MWCNTs (3-APN@MWCNT) through solvothermal grafting of 3-aminophenoxy phthalonitrile onto acid-treated carbon tubes (Figure 8a) [70]. This innovative approach achieved a remarkable 300% enhancement in *ε*_r_ (*ε*_r_ = 16.1) at 5.0 wt% loading (Figure 8c). Notably, Pu et al. engineered phthalonitrile-functionalized CNTs (CNT-CN) that react with nitrile groups on PEN backbones to form triazine-ring crosslinked structures [71]. After thermal treatment at 320 °C for 4 h, the composite achieved a record-high *ε*_r_ of 33.9 (Figure 8a,d) with exceptionally high-temperature stability.

**Figure 9:**
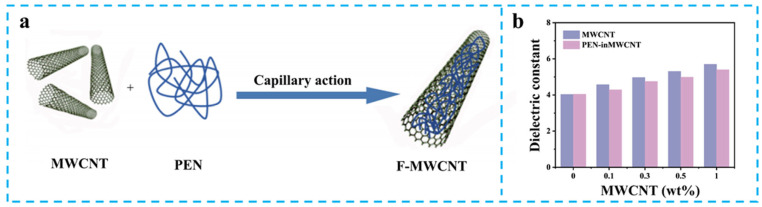
(**a**) Synthesis route of F-MWCNT; (**b**) The *ε*_r_ of F-MWCNT/PEN. Picture (**a**,**b**) were adapted with permission from Ref. [74]. Open access under the terms of the Creative Commons Attribution License (CC BY 4.0).

Structural innovation through core–shell hetero-structure design demonstrates remarkable synergistic effects. Huang et al. constructed MWCNT@BaTiO_3_ core–shell structured fillers that increased the *ε*_r_ of PEN composites to 14.2 at 1000 Hz with 50 wt% loading [72], representing a 200% enhancement over the pristine matrix [73]. Jin et al. developed a MWCNT@SiO_2_ hybrid filler system that achieved a balanced *ε*_r_ of 7.0 with a loss factor of 0.04, confirming the effectiveness of inorganic shells in suppressing conductive percolation. Particularly noteworthy is the filled MWCNT (F-MWCNT) developed by Xiao et al. [74], which leveraged capillary-induced nano-confinement effects by embedding PEN chains within the tubular cavities of MWCNT (Figure 9a). The 1.0 wt% composite system achieved a *ε*_r_ of 5.3 at 10 kHz with a low loss tangent of 0.011 (Figure 9b), establishing a new paradigm for resolving the dielectric loss paradox.

**Figure 10:**
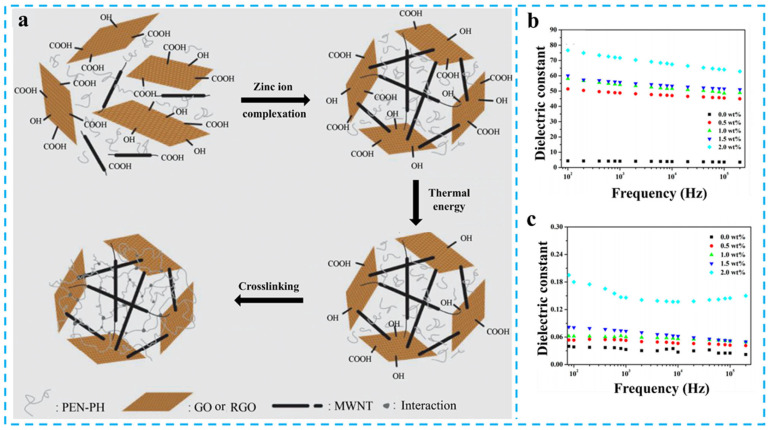
(**a**) Fabrication route (**a**), *ε*_r_ (**b**) and tan*δ* (**c**) for GS-Zn-CNT/PEN. Picture (**a**–**c**) were adapted with permission from Ref. [79]. Open access under the terms of the Creative Commons Attribution License (CC BY 4.0).

Research on graphene-based filler systems focused on interface optimization of two-dimensional structures and three-dimensional network construction [75]. Wang et al. developed reduced graphene oxide RGO/PEN composites via in situ thermal reduction of GO [76], achieving an extraordinary *ε*_r_ of 115.0 at 6.0 wt% loading (1000 Hz) with a remarkable 2300% enhancement. To address graphene aggregation issues, Li et al. functionalized GO with copper phthalocyanine (GO@CuPc), effectively suppressing the restacking of graphene sheets [77]. The 5 wt% composite exhibited a *ε*_r_ of 35. In three-dimensional architecture design, Wang et al. designed GN-Fe_3_O_4_ network, while Wei et al. constructed GS-Zn-CNT interpenetrating network demonstrating exceptional performance [78,79]. The 2 wt% GS-Zn-CNT/PEN system achieved a *ε*_r_ of 72.0 at 1000 Hz with a tan*δ* of 0.15 (Figure 10a–c), significantly lower than that of graphite/PEN nanocomposites (tan*δ* = 0.26) at equivalent 2 wt% graphene loading. This 2000% enhancement over the matrix originates from the three-dimensional network, which optimized the charge carrier migration pathways.

### 2.5. Hot-Stretching of PEN Composites

Hot-stretching, a high-efficiency polymer processing technique, remarkably enhances dielectric properties of PEN-based materials through molecular chain orientation engineering. The mechanism primarily involves three structural modulations: (1) crystallization behavior optimization via chain orientation, (2) enhanced dipolar polarization through ordered alignment of polar groups, and (3) conductive network construction via aligned nanofiller distribution. This multi-scaled synergistic effect establishes a new paradigm for designing high-performance dielectric materials.

**Figure 11:**
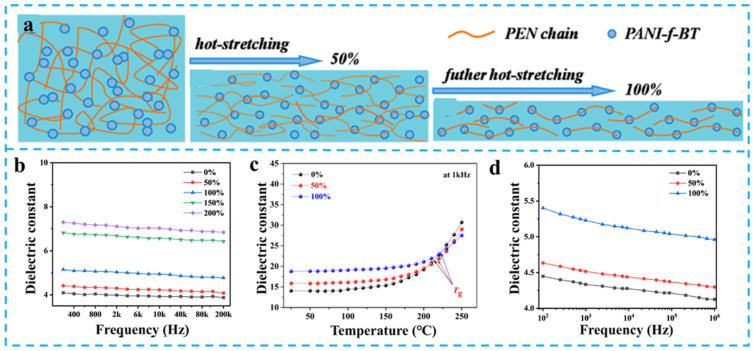
(**a**) The theoretical model of PEN composite during hot-stretching; The *ε*_r_ of PEN (**b**) [80], PANI-f-BT/PEN (**c**), and CPEN-f-BaTiO_3_@NH_2_-CuPc/PEN (**d**) [82] under different stretching ratios. Picture (**a**,**c**) were adapted with permission from Ref. [81]. Open access under the terms of the Creative Commons Attribution License (CC BY 4.0).

You et al. achieved synergistic control of molecular chain alignment and crystalline domain reconstruction through high-temperature uniaxial hot-stretching [80]. Experimental results demonstrate that 200% stretching at 280 °C elevates crystallization from 4.9% to 19.2%, accompanied by *ε*_r_ enhancement from 4.0 to 6.7 at 1 kHz (Figure 11b). Systematic investigation reveals the performance enhancement originated from a synergistic tripartite mechanism: interfacial polarization amplification via increased crystallization, dipole orientation polarization intensification through -CN alignment, and polarization response efficiency improvement by molecular anisotropy. Notably, stretching temperature critically influences crystalline integrity. The 280 °C-treated sample demonstrates superior dielectric performance (*ε*_r_ = 6.7) with lower defect density, showing 52% enhancement over the 320 °C-processed counterpart (*ε*_r_ = 4.4), which elucidates the pivotal regulatory role of thermodynamic parameters in processing.

For nanocomposite systems, hot-stretching demonstrates enhanced structural regulation advantages. In PANI-f-BT/PEN systems [81], hot-stretching treatment simultaneously aligns polymer matrix chains and induces ordered nanoparticle arrangement along the stretching direction (Figure 11a). Experimental data showed that 100% stretching elevates the *ε*_r_ from 14.0 to 18.7 at 40 wt% filler loading (Figure 11c), attributed to synergistic effects between enhanced filler–matrix interface polarization and optimized conductive pathways. You et al. further modified BaTiO_3_ nanoparticles (CPEN-f-BaTiO_3_@NH_2_-CuPc) for PEN composites [82]. After 100% hot-stretching, the system exhibited significant dielectric enhancement from 4.4 to 5.2 at 2 wt% loading (Figure 11d). This study confirms that filler surface functionalization effectively improves interfacial compatibility, while hot-stretching induced crystal nucleation strengthens filler-matrix synergism.

In conductive filler systems, Huang et al. discovered unique percolation modulation phenomena in MWCNT/PEN composites [83]. Fifty percent hot-stretching dramatically increases the dielectric constant from 105.0 to 175.0 at 1000 Hz, originating from quasi-continuous conductive networks formed by MWCNT alignment. However, excessive stretching ratio (>200%) causes filler contacting disruption and performance degradation, elucidating the dynamic equilibrium between filler dispersion state and conductive network integrity.

In summary, hot-stretching significantly enhances dielectric properties of PEN-based materials through multi-scaled structural engineering, with core mechanisms including: (1) dipole polarization enhancement via chain orientation [43], (2) interfacial polarization optimization through crystallization control, and (3) conductive network construction by aligned filler arrangement. Current research demonstrates that precise control of hot-stretching temperature, ratio [51], and filler modification strategies enables tunable dielectric performance. Nevertheless, critical scientific challenges, including high-temperature induced crystal defect evolution mechanisms and dynamic response patterns of filler networks under large strains, require further investigation, which holds significant theoretical value for developing next-generation high-performance dielectric materials.

## 3. Strategic Framework for Enhancing Breakdown Strength of PEN

This section systematically presents some major strategies for improving the breakdown strength of PEN, focusing on molecular structure design (copolymerization and thermal crosslinking), composite system construction (high-insulation filler/PEN composites), and microstructure engineering (multilayer films and thermal stretching).

### 3.1. Copolymerization Strategy

PEN, while exhibiting excellent thermal resistance and mechanical strength as a high-performance engineering plastic, suffers from limited *E*_b_ due to disordered amorphous chain arrangements, which induce electric field distortion and charge accumulation. Co-polymerization enables precise regulation of chain conformation, polar group distribution, and crystallization behavior, effectively optimizing the structure-property synergy in dielectric materials [84]. This subsection focuses on co-polymer modification strategies, elucidating enhancement mechanisms through chain rigidification design and polar group functionalization [32,85], providing theoretical guidance for designing high-energy-density dielectric materials.

**Figure 12:**
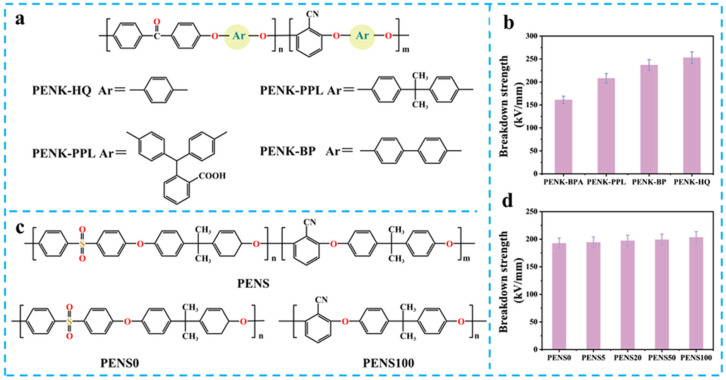
(**a**) Structures of PENK copolymers [86]; (**b**) the *E*_b_ of the PENK copolymers [86]; (**c**) structures of PENS copolymers [87]; (**d**) the *E*_b_ of the PENS copolymers [87].

Chain rigidification and ordered alignment constitute the core strategy for *E*_b_ enhancement. Mao et al. synthesized poly(arylene ether nitrile ketone) (PENK) copolymers via nucleophilic aromatic substitution co-polymerization using 2,6-dichlorobenzonitrile (DCBN), 4,4′-difluorobenzophenone (DFBP), and various bisphenol monomers (HQ, BP, BPA, PPL) (Figure 12a) [86]. The study revealed that PENK-HQ and PENK-BP with rigid HQ/BP monomers form partially crystalline structures due to enhanced backbone regularity, achieving *E*_b_ of 253 kV/mm and 237 kV/mm significantly surpassing their amorphous counterparts (PENK-BPA: 161 kV/mm; PENK-PPL: 208 kV/mm) (Figure 12b). According to Tanaka’s charge injection theory, crystalline regions reduce trap density through ordered chain alignment, suppressing charge injection and space charge accumulation, thereby retarding electrical tree growth. DSC thermal analysis confirms this mechanism: PENK-HQ and PENK-BP exhibit melting enthalpies of 0.96 J/g and 1.50 J/g, respectively, where increased crystallization suppresses charge transport under high electric fields.

For polar groups synergistic modulation, Hu et al. constructed poly(arylene ether nitrile sulfone) (PENS) (Figure 12c) systems via ternary co-polymerization of DCBN, bisphenol A (BPA), and bis(4-chlorophenyl) sulfone (BCPS). As the PEN segment content increases from PENS0 to PENS100 [87], the gradual elevation of cyano group density progressively enhances *E*_b_ from 192 kV/mm to 203 kV/mm (Figure 12c). Mechanistic studies revealed that the high electronegativity of cyano groups enhances local polarization, while the rigid benzene-sulfone structure restricts chain segment mobility and reduces trap-state density. DSC analysis showed single glass transition temperatures (*T*_g_ ≈ 180–210 °C) for all PENS copolymers, confirming molecular chain homogeneity. This phase-separation-free microstructure minimizes internal defects, substantially reducing electric field distortion risks.

This subsection analyzes the enhancement mechanisms of co-polymerization strategies on PEN breakdown strength. Through chain rigidification design, polar group functionalization, and dynamic crosslinking network construction, researchers successfully increased PEN-based material *E*_b_ from 161 kV/mm to 253 kV/mm, achieving a 57% enhancement.

### 3.2. Thermal Crosslinking Strategy

Thermal crosslinking has garnered increasing attention due to its unique capability for constructing three-dimensional (3D) network architectures. This technique establishes 3D crosslinked networks through covalent bonding of molecular chains, which not only remarkably enhances thermal stability but more regulates charge carrier transport behavior and suppresses space charge accumulation, thereby synergistically optimizing *E*_b_ and *U*_e_ [88]. A pioneering design was developed by Wei et al. through blending phthalonitrile-functionalized titanium dioxide nanoparticles (TiO_2_-CN) with phthalonitrile-terminated PEN (PEN-Ph) [89]. Subsequent thermal crosslinking at 320 °C for 4 h successfully yielded TiO_2_-PEN hybrid materials. The resultant system demonstrated substantially improved thermostability with *T*_g_ elevated to 229 °C and 5% thermal decomposition temperature (*T*_5%_) exceeding 524 °C, establishing fundamental characteristics for high-temperature energy storage applications. The crosslinked network optimized dielectric properties through dual synergistic mechanisms: (1) The three-dimensional network effectively suppressed molecular chain relaxation, reducing tan*δ* to 0.008 at 1000 Hz; (2) physical confinement effects diminished free volume and restricted charge carrier migration pathways. The research team extended this strategy by incorporating functionalized boron nitride (BN-2CN) and carbon nanotubes (CNT-2CN) into the PEN matrix. High-temperature self-crosslinking produced CPEN-BN-CNT ternary hybrid materials, achieving a breakthrough *E*_b_ of 275 kV/mm [90], which experimentally validated the feasibility of multi-component synergistic enhancement.

**Figure 13:**
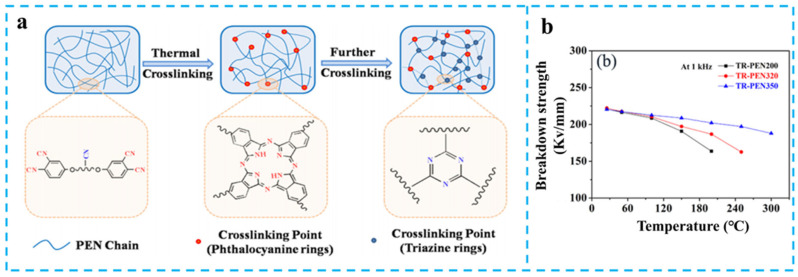
(**a**) Scheme for the fabrication of the TR-PENs; (**b**) The *E*_b_ of the TR-PENs. Picture (**a**,**b**) were adapted with permission from Ref. [91]. Copyright 2019 American Chemical Society.

Precise modulation of crosslinking density proves pivotal in optimizing high-temperature material performance. You et al. engineered crosslinking density gradients in the crosslinkable TR-PEN200 system via stepwise thermal processing (320 °C/350 °C) [91], fabricating TR-PEN320 and TR-PEN350 with distinct crosslinking densities (Figure 13a). Electrical characterization revealed a marginal difference in *E*_b_ at room temperature, while exhibiting a pronounced gradient at 180 °C: TR-PEN200 (165 kV/mm) < TR-PEN320 (191 kV/mm) < TR-PEN350 (208 kV/mm, Figure 13b). This temperature-dependent behavior originates from dual competing mechanisms: On one hand, elevated temperature intensifies chemical bond vibrations and migration of impurities within the polymeric matrix, thereby elevating electrical conductivity and thermal breakdown susceptibility; on the other hand, densely crosslinked networks effectively constrain molecular chain mobility and suppress long-range charge carrier migration under high temperature. Experimental evidence confirmed that beyond critical crosslinking density, the spatial confinement effect of network architectures dominates dielectric behavior, resulting in a 26% enhancement for *E*_b_ at high temperatures.

Thermal crosslinking offers multi-scaled optimization pathways for enhancing dielectric energy storage in PEN: (1) Significantly reinforced thermomechanical stability through 3D covalent network construction [92], (2) reduced dielectric loss via steric hindrance effects that impede charge carrier migration, (3) Optimized electric field distribution through free volume modulation [93].

### 3.3. High-Insulation Filler/PEN Composites

As a representative 2D wide-bandgap insulator (*E*_b_ > 800 kV/mm, bandgap ~6.0 eV), boron nitride nanosheets (BNNS) with a unique lamellar structure emerge as ideal candidates for optimizing breakdown strength of PEN [94,95,96]. Early-stage investigations primarily focused on single-filler reinforcement effects of BNNS. Pu et al. fabricated BNNS with lateral dimensions of ~1.5 μm via liquid-phase ultrasonic exfoliation [97]. At 12 wt% loading, the BNNS/PEN composite exhibited a breakthrough in *E*_b_ from 180 kV/mm (pristine matrix) to 240 kV/mm, representing a 33.3% enhancement. This remarkable improvement originates from triple synergistic mechanisms of BNNS: (1) formation of percolative 2D networks for electric field homogenization [98], (2) interlayer high potential barriers suppressing electron tunneling [99], (3) in-plane high thermal conductivity (~400 W/mK) facilitating rapid joule heat dissipation [100]. Based on this foundation, Lan et al. innovatively engineered h-BN/GO hybrid fillers, constructing hetero-interfaces through π-π stacking interactions. At 7.5 wt% h-BN-GO loading, the composite achieved 91.6% higher *E*_b_ than pure GO/PEN systems [101]. This originates from localized potential wells induced by space charge polarization at heterointerfaces, which effectively trap charge carriers and reduce leakage current density.

To overcome nanofiller agglomeration challenges, He et al. developed a core–shell architecture strategy [102], fabricating BPh-microsphere@BNNS composites via electrostatic self-assembly. The steric hindrance effect from the microsphere enabled uniform BNNS-OH dispersion, achieving 242 kV/mm *E*_b_ at merely 4.4 vol% loading, representing 34.2% improvement over pristine PEN. Three-dimensional tomography revealed graded dielectric interfaces within the matrix, generating multilevel trap states via Maxwell–Wagner polarization, thereby reducing charge carrier mobility. Wei et al. implemented molecular-scale engineering through cyano-functionalization to create BN-CN fillers [90]. At 8 wt% BN-CN content, *E*_b_ surged to 318 kV/mm (40.8% enhancement), setting the current record for PEN-based composites. The functionalization treatment elevated interfacial binding energy, effectively suppressing electrical treeing propagation.

The aforementioned studies demonstrate that multidimensional filler engineering design can significantly enhance *E*_b_ in PEN composites. This progression spans from electric field regulation via single BNNS fillers [103], through interfacial polarization enhancement with hybrid fillers [104], to molecular-level interface reinforcement through core–shell architectures and chemical functionalization, illustrating an evolutionary trajectory from macroscopic construction to in-depth microscopic mechanism exploration [105,106].

### 3.4. Multilayer Films

Among the PEN-based dielectric energy storage materials, pinhole defects pose critical challenges to *E*_b_. Studies revealed nanoscale pinholes in solution-cast PEN films, present on both surfaces and cross-sections, arising from the high molecular weight characteristics of polymer chains during solvent evaporation and being inherently irremovable [107,108,109,110]. Air-filled pinholes with dielectric strength substantially lower than the polymer matrix severely degrade overall breakdown performance. To address this problem, Tang et al. proposed a bi-layered stacking strategy: laminating two 20 μm-thick PEN films using high-temperature silicone oil (0.3 μm-thick) as adhesive [111]. Experimental results demonstrated that the bi-layered structure achieves ‘pinhole shielding effect’ where defect-free regions in the upper layer physically mask pinholes in the lower layer, effectively mitigating electric field concentration and localized breakdown risks. Eventually, this architecture elevated *E*_b_ to 364 kV/mm. In contrast, biaxially stretched BOPP films inherently avoid pinhole defects due to their melt manufacturing process [112,113,114], rendering the bi-layered configuration less effective for *E*_b_ enhancement, thereby highlighting the unique advantage of multi-layered design in optimizing the breakdown strength of PEN.

### 3.5. Hot-Stretching of PEN Composites

**Figure 14:**
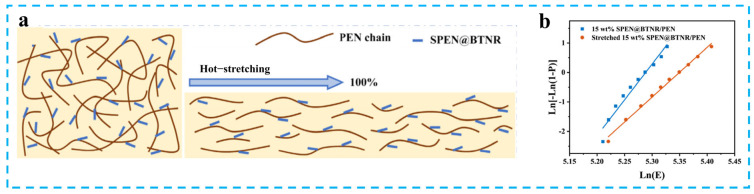
(**a**) Hot−stretching mechanism diagram; (**b**) *E*_b_ of 15 wt% SPEN@BTNR/PEN nanocomposite films before and after hot-stretching. Picture (**a**,**b**) were adapted with permission from Ref. [59]. Copyright 2024 Springer Nature.

The efficacy of hot-stretching in enhancing *E*_b_ of PEN-based composites has been rigorously validated across multiple material systems. This technology’s fundamental breakthrough lies in achieving filler alignment through synergistic thermo-mechanical action, effectively mitigating localized electric field distortion [115,116,117]. Zhang et al. pioneered sulfonated PEN (SPEN)-functionalized barium titanate nanorods (SPEN@BTNR), which were aligned along the stretching direction within the PEN matrix via a hot-stretching process (stretching ratio 100%, 200 °C) [59], forming a nacre-like layered architecture (Figure 14a). This oriented structure enabled SPEN@BTNR/PEN composite to maintain an exceptional *E*_b_ of 210 kV/mm at 15 wt% high filler loading (Figure 14b). Gao et al. and Liu et al. further elucidated the strengthening mechanisms of hot-stretching on breakdown behavior via employing SPEN-modified calcium copper titanate nanorods (SPEN@CCTONR) and cyano-functionalized PEN (CPEN)-modified barium strontium titanate nanorods (CPEN@BSTNR), achieving high *E*_b_ of 199 kV/mm and 204 kV/mm at 15 wt% and 16 wt% filler loading after hot-stretching [118,119]. These findings collectively demonstrate that hot-stretching technology induces nanofiller alignment, effectively alleviating electric field distortion caused by high *ε*_r_ fillers [120,121], thereby providing an innovative solution for enhancing *E*_b_ in PEN-based composites.

## 4. Conclusions and Perspectives

This work systematically summarizes recent breakthroughs in multiscale structural regulation strategies for enhancing the energy storage performance of PEN-based dielectric composites. Building on the strong polarity and modifiable characteristics of cyano groups in PEN molecular chains, we innovatively propose a trinity synergistic strategy encompassing ‘molecular polarization regulation-dielectric synergy design-microstructure engineering’. Studies have demonstrated that the integration of organic/inorganic multiphase composite system design with uniaxial hot-stretching post-treatment enables synergistic optimization of relative *ε*_r_ and *E*_b_. Despite breakthroughs in energy density, significant attenuation in energy conversion efficiency remains a critical barrier for practical applications in high-voltage power equipment. Therefore, developing PEN-based composites with concurrently high energy density and efficiency emerges as a pivotal challenge requiring urgent resolution.

Future research should focus on three strategic dimensions: First, in interfacial engineering, priority should be given to developing gradient core–shell fillers with self-adaptive dielectric properties. Compared with conventional SiO_2_@PEN systems, MXene-based hetero-junction structures may achieve local electric field redistribution through interfacial polarization regulation. Density functional theory (DFT) calculations enable precise prediction of dipole interaction intensity between filler surface functional groups and PEN cyano groups, providing theoretical guidance for constructing low-loss interfacial layers. Second, in terms of filler dimensional engineering, the construction of a multi-component topological structure incorporating 2D nanosheets and 3D nanoflowers can synergistically enhance both the polarization response efficiency and energy storage density of the PEN matrix. Finally, in device integration, adopting vapor deposition techniques from semiconductor packaging to develop all-organic composite dielectric films with 3D interpenetrating network structures is recommended. This biomimetic layered architecture can synergistically enhance mechanical flexibility and high-temperature energy storage stability.

Notably, recent breakthroughs in bio-based poly(ether nitrile) synthesis have opened new avenues for developing environmentally friendly dielectric materials. Covalent grafting of lignin derivatives with PEN molecular chains not only improves dielectric anisotropy but also substantially reduces raw material costs. We anticipate that the convergence of computational materials science, intelligent manufacturing technologies, and novel synthesis methods will propel PEN-based dielectric materials with superior energy density and cycling stability toward widespread applications in smart grids and new energy vehicles.

## Data Availability

Not applicable.
